# A Serious Game to Self-Regulate Heart Rate Variability as a Technique to Manage Arousal Level Through Cardiorespiratory Biofeedback: Development and Pilot Evaluation Study

**DOI:** 10.2196/46351

**Published:** 2023-08-24

**Authors:** Tony Estrella, Carla Alfonso, Juan Ramos-Castro, Aitor Alsina, Lluis Capdevila

**Affiliations:** 1 Laboratory of Sport Psychology Department of Basic Psychology Universitat Autónoma de Barcelona Barcelona Spain; 2 Sport Research Institute Universitat Autònoma de Barcelona Barcelona Spain; 3 Group of Biomedical and Electronic Instrumentation Department of Electronic Engineering Universitat Politècnica de Catalunya (UPC) Barcelona Spain; 4 Department of Information and Communications Engineering Universitat Autònoma de Barcelona Barcelona Spain

**Keywords:** serious game, heart rate variability, biofeedback, mobile health, mHealth, app, mobile phone

## Abstract

**Background:**

Heart rate variability biofeedback (HRVB) is an established intervention for increasing heart rate variability (HRV) in the clinical context. Using this technique, participants become aware of their HRV through real-time feedback and can self-regulate it.

**Objective:**

The aim of this study was 2-fold: first, to develop a serious game that applies the HRVB technique to teach participants to self-regulate HRV and, second, to test the app with participants in a pilot study.

**Methods:**

An HRVB app called the FitLab Game was developed for this study. To play the game, users must move the main character up and down the screen, avoiding collisions with obstacles. The wavelength that users must follow to avoid these obstacles is based on the user’s basal heart rate and changes in instantaneous heart rate. To test the FitLab Game, a total of 16 participants (mean age 23, SD 0.69 years) were divided into a control group (n=8) and an experimental group (n=8). A 2 × 2 factorial design was used in each session. Participants in the experimental condition were trained in breathing techniques.

**Results:**

Changes in the frequency and time domain parameters of HRV and the game’s performance features were evaluated. Significant changes in the average RR intervals and root mean square of differences between adjacent RR intervals (RMSSD) were found between the groups (*P*=.02 and *P*=.04, respectively). Regarding performance, both groups showed a tendency to increase the evaluated outcomes from baseline to the test condition.

**Conclusions:**

The results may indicate that playing different levels leads to an improvement in the game’s final score by repeated training. The tendency of changes in HRV may reflect a higher activation of the mental system of attention and control in the experimental group versus the control group. In this context, learning simple, voluntary strategies through a serious game can aid the improvement of self-control and arousal management. The FitLab Game appears to be a promising serious game owing to its ease of use, high engagement, and enjoyability provided by the instantaneous feedback.

## Introduction

### Background

Heart rate variability (HRV) represents the temporal variation between successive heartbeats (RR interval), and it is measured in milliseconds [1]. HRV is a biomarker of cardiovascular [2] and mental [3] health, reflecting the balance between the parasympathetic and sympathetic branches of the autonomic nervous system (ANS) [4]. HRV has been considered as an objective and noninvasive health indicator, with higher resting HRV being associated with lower cardiovascular risk [5], reduction of negative emotions through acute stress [6], flexible and adaptive response to environmental demands [7], and improved fitness [8]. This reflects the numerous applications of HRV in various fields.

HRV biofeedback (HRVB) is an established intervention that increases HRV in the clinical context and has proven to be effective as a treatment for anxiety and depression [9,10]. With this technique, participants become aware of their HRV through real-time feedback and can self-regulate it, restoring its homeostasis [11]. HRVB is based on the physiological phenomenon of respiratory sinus arrhythmia, which is defined as the variation in heart rate (HR) caused by respiration [12]. The analysis of respiratory sinus arrhythmia shows that the instantaneous HR (iHR) increases during inhalation and decreases during exhalation. Thus, the original HRVB procedure proposed by its promoters emphasizes the importance of breathing training to maximize HRV and achieve the maximum benefits of this technique [12]. In a recent systematic review [13], a series of methodological guidelines were proposed to improve the effectiveness of HRVB interventions. This review highlighted the importance of the breathing protocol as the core of the HRVB interventions, concluding that concrete aspects such as the inhalation, holding, or exhalation ratio should be explained in detail to the participants who apply the technique. Furthermore, HRVB can help establish a connection between the mind and body through voluntarily controlled physiological behaviors, such as breathing, facilitating the learning process and self-control.

HRVB is becoming more widely used owing to its ease of application and high cost-benefit potential, particularly because of the current accessibility of health technologies through mobile health devices [14,15]. The growth of mobile health technology has allowed the development of game-based mobile apps, which are known to increase the transfer of learning and motivation toward practice [16]. The use of games either in health or educational interventions allows the participants to immerse themselves in the activity that is required by the game, promoting a flow state [17,18]. Similarly, other studies have shown that when playing serious games, the student’s role changes from a passive receiver of information to an active entity interacting with the game, facilitating the learning process [19,20]. Serious games, rather than being geared toward entertainment, are aimed at educating, training, and informing the player about a specific topic [21]. They are being used in various health domains [22-24]. For instance, in the field of HRVB, they can be a way to teach participants to control HRV through breathing techniques in an interactive environment.

### Objectives

With the information provided in the *Background* section, the aim of this study was 2-fold: first, to develop a serious game that applies the HRVB technique to teach participants to self-regulate HRV and, second, to test the app with participants in a pilot study.

## Methods

This study has two phases: (1) the development of a serious game biofeedback app called the FitLab Game and (2) the testing of the app with participants in a pilot study.

### Phase 1: App Development

#### Visual Environment and Game Explanation

##### General Design

In accordance with definition of serious games by Djaouti et al [25], the FitLab Game is meant for physical and mental training in the health care sector and intended for the general public. The overall goal of the game is to help the user self-regulate their HRV by providing biofeedback in an attempt to follow or mimic a preset iHR waveform. The architecture and design of the game allows the incorporation of new real iHR curves (RR) to imitate and can be adapted to different levels of complexity in the different existing scenarios (levels). These real recordings can be chosen by the player or a professional accompanying the player. Depending on the level and the preset iHR waveform chosen, the game will lead to a state of relaxation (increasing cardiac variability) or a state of activation in the participant (decreasing cardiac variability), according to the needs demanded by each particular situation. Different gamification techniques were used in its design to increase the players’ engagement with the tasks. More general techniques include having a goal to aim toward, a set of rules to follow, a challenge to complete, and *real*-*time* feedback to tell the player how he is doing and how close he is to completing the goal. More specific gamification techniques were added to further engage the player, including Bonus points, increasing challenge, and performance graphs.

##### Menus

**Figure 1:**
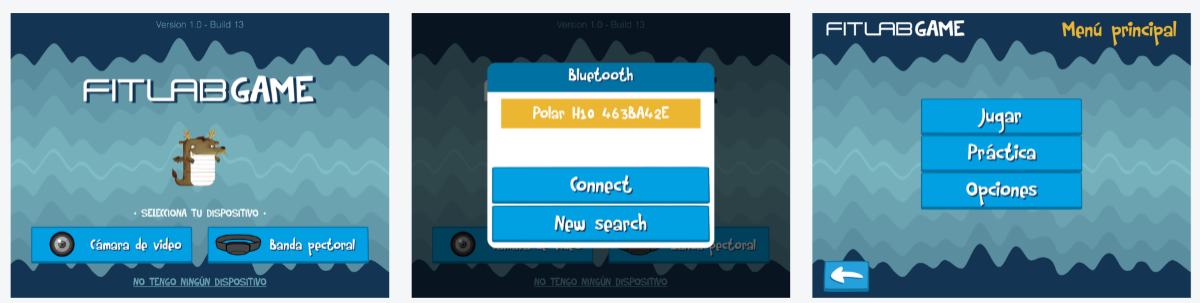
Screenshots of the game’s menus. Game’s transition from the main screen to the screen that appears when you select “Banda pectoral” (“Chest Band”) and which allows to connect a heart rate sensor to the game. On the right, the third menu shows where the player can select “Jugar” (“Play”) to play.

The game’s menus were designed in landscape mode and presented in Spanish. The first screen that appears when opening the app is the main screen, which allows users to choose the sensor used to input data: either the device’s camera (“Cámara de vídeo”) or a HR band (“Banda Pectoral”). The latter was used in this study. Once connected to the sensor, the app allows access to the game’s mode, where the player can select between the option “Jugar” (“Play”), in which the user overcomes different levels in an orderly manner so that the difficulty of the game gradually increases, or the option “Práctica” (“Practice”), in which the user can activate an independent session. The tab “Opciones” (“Options”) refers to the game’s settings (Figure 1).

##### Screens

**Figure 2:**
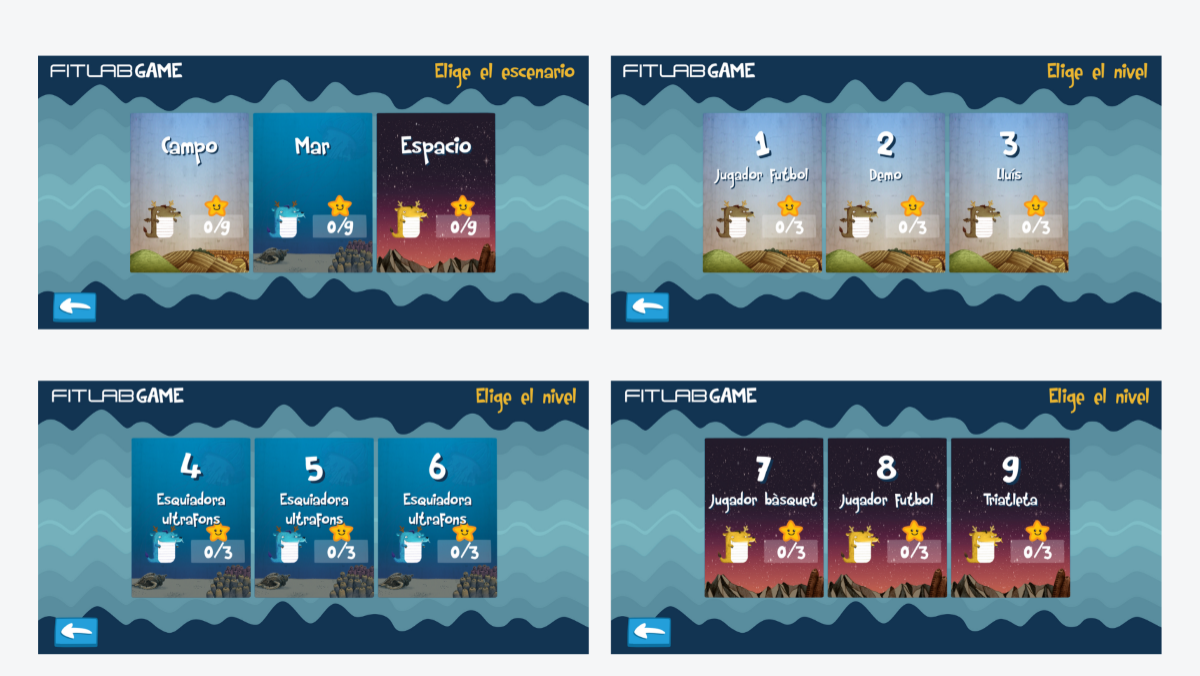
Screenshots of worlds and scenarios of the game: “Campo” (“Rural area”), “Mar” (“Sea”), and “Espacio” (“Space”).

The FitLab Game has 9 levels distributed in 3 visually different worlds (Figure 2). Each world has 3 scenarios or levels of difficulty that the user must overcome in a set order to be able to access the next one when in “Jugar” (“Play”) mode. To overcome a scenario, the user must move the main character up and down the screen to avoid colliding with obstacles that keep appearing. Touching an obstacle results in the loss of a life, and losing all lives means the game is over and needs to be restarted. The movement of the main character reflects cardiac variability and can be controlled by different voluntary strategies such as breathing.

**Figure 3:**
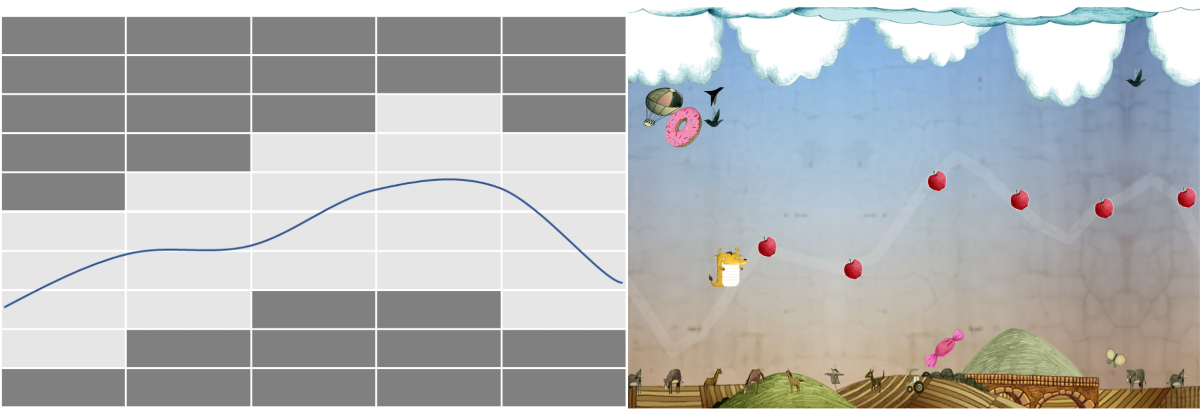
Example of wave based on an instantaneous heart rate curve from a particular RR interval series.

The upper and lower limits and the obstacles in each scenario of the game are not placed randomly. They follow a specific waveform determined by previously recorded RR intervals, corresponding to a real recording. Each scenario represents a real RR series transformed and visualized as iHR, with different difficulty to imitate depending on the particular and distinct RR series (represented as a curve of iHR values) on which each scenario is based. Before starting the game, the data were calibrated to establish an initial HR range for the user (based on iHR from RR intervals). An algorithm was created specifically for the game, which takes this initial HR and displays it on a “grid” (invisible to the user) where each box is 1 second wide per 1 second high. With this algorithm, the screen is configured to place the character in the central area vertically, and the width of the screen is adjusted such that it is proportional to the range of variability presented by the user. In addition, the upper and lower limits and the obstacles, based on the previously recorded RR series, are placed above and below the defined waveform, leaving a space for the character to pass in between (Figure 3).

As a result of the initial calibration, all users start the game under similar conditions, while also having the level of difficulty of each scenario of the game adjusted to their own HR values. Overall, the configuration of each game is a dynamic and individual process, setting it apart from “one-size-fits-all” games. The individually tailored nature of the FitLab Game was designed to increase motivation and the learning process [26].

**Figure 4:**
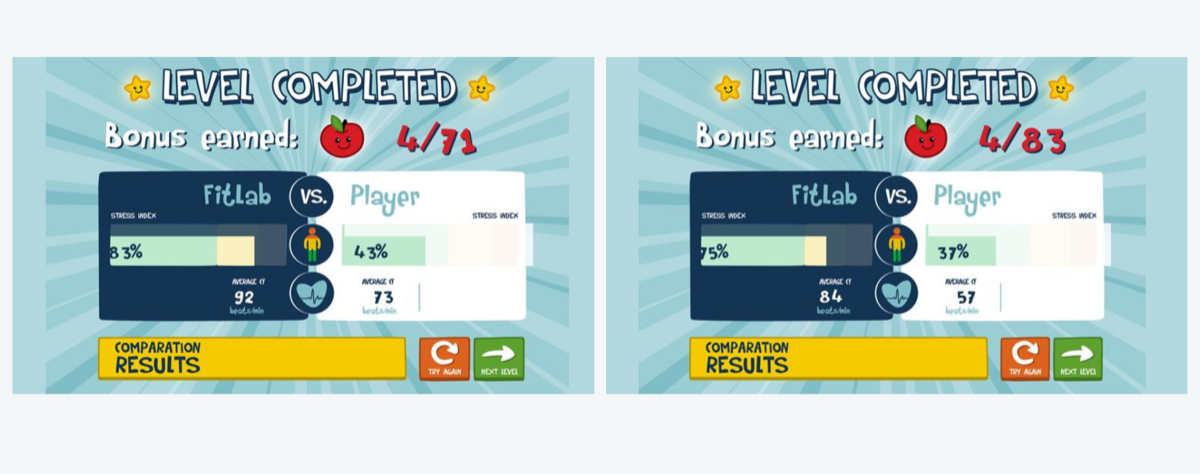
Example of the final page, appearing after a word of the FitLab Game is played.

The left side of Figure 3 illustrates the algorithm that defines the ideal pathway of the character. The blue line represents the waveform defined by the prerecorded iHR curve (from a specific RR series), and the dark gray boxes represent the upper and lower limits of this curve, where the obstacles would be positioned. The right side of Figure 3 shows an example of how this algorithm would be seen in the game (see a dynamic example in Multimedia Appendix 1). Once the obstacles are placed, the objects and landscape of the scene are selected. For each world, there is a bank of images that can be used (mountains, animals, houses, etc) and placed by distributing them according to their size. Larger objects form the background plane, such as the mountains. Medium objects, such as houses, gradually fill the spaces that the first type of images could not fill because of their size, and small objects, such as birds, trees, or other details, are placed in the spaces that are missing to be filled in, which in Figure 3 are the dark gray boxes. Notably, the placement of obstacles is not exhaustive, meaning that it is not intended to fill all the space reserved for obstacles; otherwise, the screen will be saturated with objects. The goal is to place objects in the most harmonious way possible but clearly marking the path that the main character should follow. When the user reaches the end of the level or loses a life owing to touching obstacles, a final page appears. On this screen (Figure 4), a “Level Completed” message is shown, together with the total points collected, and the average stress index and average HR of the player and of all participants who have played the game is also shown.

#### App Architecture

##### Programing Language and Functionalities

The architecture of the app consists of two parts: (1) the base of the game and (2) the container of the app and the communication with another device. The base of the game refers to the visual part, the game’s engine, screen management, menus, and navigation. This entire part has been implemented with HTML (WHATWG) assisted by CSS (World Wide Web Consortium) and JavaScript (Oracle Corporation) technologies, so that it can be run in any web browser, whether hosting the application on a web server or running it directly on a local computer or mobile device. Note that opening the game in a web browser will not allow the use of the sensors and hardware; therefore, on a web browser, the game can only run in the test mode. Nonetheless, in the test mode, it is still fully functional using the cursor on a computer keyboard or by tapping on the screen of a mobile device, which allows testers to move the character up and down the screen manually.

The container refers to what allows the game to be installed as a mobile app on an Android or iOS operating system. In addition, it provides the necessary functionality to access the camera of the device and process the images as well as to establish Bluetooth connections with HR sensors and process the collected data. In this case, data processing is used in the game as input commands, replacing the usual input methods, including the keyboard or touch screen (for testing purposes). That is, it is with iHR data (from real-time RR intervals) that the user can move the main character up and down the screen using the variation of each iHR beat with respect to the previous one. This app container was implemented using a cross-platform environment for writing applications for Android and iOS operating systems called Titanium SDK (TiDev, Inc).

**Figure 5:**
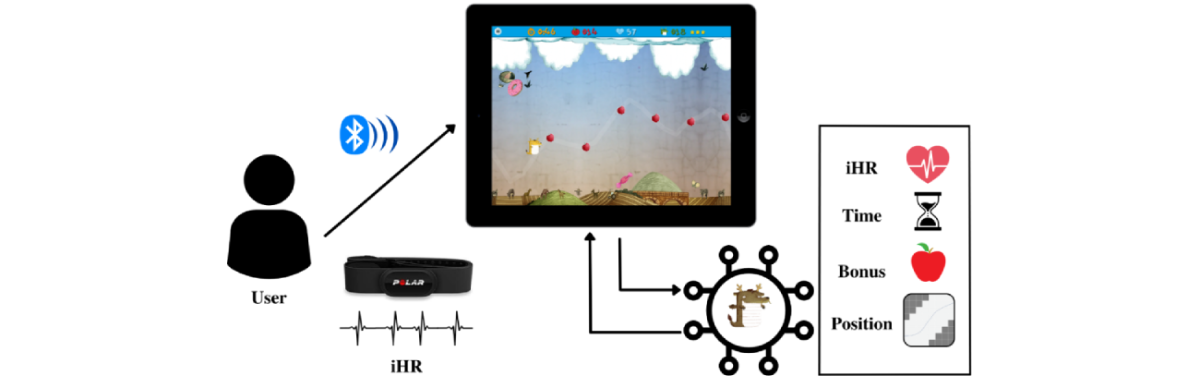
Game flow representation. iHR: instantaneous heart rate.

The game engine is written in JavaScript and is based on a constant loop where, at each step, the current position of the character is evaluated with respect to its environment (screen boundaries, obstacles, and prizes) and the input of the user (iHR data; Figure 5). The game engine, menu screens, and navigation are written in web languages compatible with any browser. Finally, both operating systems (Android and iOS) allow the introduction of web components within the app to reproduce web code (ie, HTML+CSS+JavaScript) as if it were part of the same app. The current app takes advantage of this feature to separate the game from the app’s container, which allows communication via Bluetooth with other devices. When the container receives a command via Bluetooth, the app container code sends the corresponding interpretation of this command to the game part. For example, when instantaneous HR data are received over Bluetooth, the app container unpacks the received frame, obtains specific iHR data, and sends it to the game, which interprets it and moves the character. Figure 5 shows a game flow representation, where the user wearing a chest band sends iHR signals via Bluetooth and the game algorithm evaluates iHR, time playing, Bonus achieved, and position on a constant loop.

##### Data Collection and Bluetooth Connection

Before communication occurs between the sensor (HR sensor) and the FitLab Game, a connection must be established between the entities. Such a connection is established via Bluetooth and is known as pairing. In this type of connection, one of the devices acts as a host or receiver of data and the other acts as a client or sender. The device acting as a receiver can establish connections with multiple sending devices, but a sending device can only connect to a single receiver. Currently, the present app allows only 1 sender to be connected to the device because each game is calibrated to a user’s iHR.

It should be noted that the iHR reading device used for the FitLab Game could be any, but it must offer a Bluetooth low energy connection so that the app can obtain the data. This version of Bluetooth can define specific data formats using standard attribute profiles (Generic Attribute Profile). This allows, for example, Bluetooth communication in the Heart Rate Profile mode to be established between the data sensor (the sender) and the app (the receiver). In this way, the receiver can interpret the data in whichever format it is packaged and can obtain the iHR information regardless of the manufacturer and model of the sending device, as long as it uses Bluetooth low energy with the Heart Rate Profile mode.

At present, when the app is opened, the user is asked to select the data entry method. When Bluetooth is selected, a screen with detected nearby Bluetooth devices is displayed. The list of displayed devices is filtered to show only those in the Heart Rate Profile mode. The user can select a device from the list of available devices and establish a connection. This process may take 1 or 2 seconds, and the user receives a confirmation if the connection has been successfully established or if an error has occurred. Once the connection is established, the application takes the user to the game’s playing menu.

##### Data Processing

For each game carried out in the app, iHR data are collected together with other metadata, including the start and end times of the game, a user’s identifier code, an ID assigned to the session, the level of the game played, the Bonus achieved, and the iHR calibration. All these data are packaged, saved in the container of the app, and can later be exported for analysis. Multiple user sessions can be stored locally, that is, in the application, but an internet connection is required to export the data to a server. Note that the game does not need an internet connection to work, and a wireless Bluetooth connection between the sender and the receiver in proximity is sufficient. For research purposes, the sessions that are temporarily saved on the device are accessible from a configuration menu by an application manager. To export data, the manager needs to select the desired sessions and indicate an email address to which the anonymized data will be sent, that is, identified by user codes and never by names or other identifying data.

### Phase 2: Pilot Study

#### Study Sample

A total of 16 participants (9 men and 7 women; mean age 23, 0.69 years) were divided into 2 groups: a control group (4 men and 4 women; mean age 23.25, SD 0.99 years) and an experimental group (5 men and 3 women; mean age 22.75, SD 1.01 years). All participants were aged >18 years and did not present any cardiovascular disorder or take any medication that could affect HRV. The descriptive statistics of the participants are presented in Table 1. All participants, selected via a convenience sample of university students, were volunteers, and all participants provided written informed consent.

**Table 1:** Descriptive data of the participants (N=16).

Variable	Control group (n=8)	Intervention group (n=8)	All participants (N=16)	*P* value
Gender (woman), n (%)	4 (50)	3 (38)	7 (44)	.61
Age (years), mean (SD)	23.25 (2.82)	22.75 (2.87)	23 (2.76)	.73
Height (cm), mean (SD)	170 (10.53)	173.88 (8.34)	171.94 (9.39)	.43
Weight (kg), mean (SD)	66.5 (13.86)	66 (9.41)	66.25 (11.45)	.93
BMI (kg/m^2^), mean (SD)	22.84 (3.38)	21.78 (2.50)	22.31 (2.92)	.49

#### Material and Instruments

##### Control Measures Self-Report

Before attending the session, participants were recommended, by email, to avoid taking nonessential drugs (up to 24 hours before the session) as well as caffeine, smoking, or any other psychostimulant (up to 2 hours before); alcohol (10 hours before); heavy meals (3 hours before); or eating in general (1 hour before). They were also asked to avoid high-intensity physical activity or any unusual exercise (20 hours before), to sleep for at least 6 hours, and to wear comfortable clothes to the laboratory. A brief questionnaire was administered immediately before the laboratory sessions to record these conditions. The participants were also asked to report their weight and height. In addition, the BMI was computed based on the participants’ reported height and weight. This measure was used as a control variable between the groups because of its possible influence on HR [27].

##### Polar Band H10

An H10 cardiac chest band (Polar Electro) was used to record the RR interval signal (transformed to iHR), as validated previously [28]. The signal was sent and collected to the FitLab Game App (iOS version 1.0, build 1; Health&SportLab SL), which was active on an iPad Air 2 (version 12.1.4).

##### Questionnaire of Satisfaction With FitLab Game

An ad hoc satisfaction questionnaire based on the proposals of different works [29-31] was tailored to our research purpose. The questionnaire was created to collect participants’ information about the following factors (see *item number* in brackets): (1) usefulness, (2) ease, (3) enjoyment of the game, (4) personal ability, (5) intention to use, (6) clarity of the goal of the game, (7) controllability of the game, (8) strategic approach, (9, 10, and 11) flow, (12) feedback, (13) educational aspect, (14 and 15) motivation, and (16) design aspects. Each factor was represented by a single item, except for flow and motivation, which consisted of 3 and 2, respectively. The participants responded to the questionnaire using a Likert scale ranging from 1 (strongly disagree) to 5 (strongly agree). Mean scores >3 can be considered as positive, 3 as neutral, and <3 as negative. Items related to the techniques used to modulate HR were not included in the control group. The average total score was computed based on participants’ responses, providing a general satisfaction score.

#### Procedure

**Figure 6:**
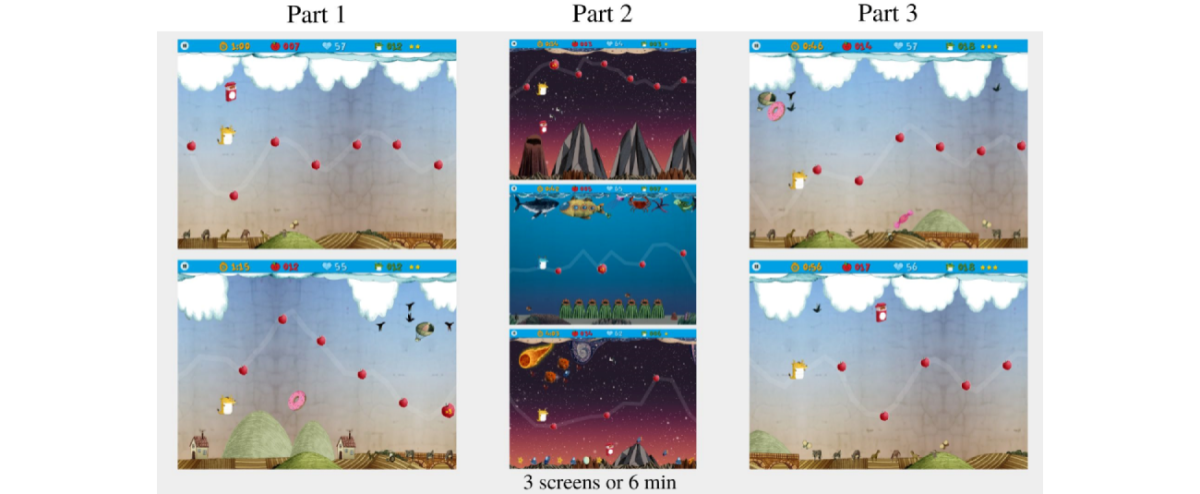
Example of screens of the game that participants played. Testing consisted of 3 parts (part 1: baseline phase, part 2: practice phase, and part 3: test phase).

The study consisted of 1 individual session per participant, which lasted for approximately 35 minutes and took place in the Basic Psychology Laboratory of the Universitat Autònoma de Barcelona. The day before this session, each participant was provided a list of recommendations, based on the *Control Measures Self-report****,*** to record variables that may affect HRV, including physical activity and intake of caffeine, psychostimulants, alcohol, drugs, and food. At the beginning of the session, the H10 cardiac band was placed on the participant’s chest so that they could become familiarized with the sensation of the band. A few drops of conductive gel were applied to the band to facilitate good contact with the skin. Once the band was placed, the participant answered the *Control Measures Self-report* questionnaire. The participants were then introduced to the FitLab Game for the first time. Play time was divided into 3 parts, with the first and third parts being recorded for the study (Figure 6). In part 1, the baseline phase, each participant played 2 levels of the game to familiarize themselves with the functioning of the app, without time constraints. In part 2, the practice phase, participants practiced on a different level for 6 minutes, or a maximum of 3 levels. Finally, in part 3, the test phase, each participant played the same 2 levels as in part 1.

A few techniques to consciously control iHR (and thus manage HRV) were explained to the participants in the experimental group but not to those in the control group. To increase HRV (and reduce iHR), it was recommended to either breathe at a pace where the exhalation was longer than the inhalation [32-34] or to hold one’s breath [35]. To reduce HRV (and increase iHR), participants were instructed to slightly hyperventilate [36], to perform muscle movements [37], or to swallow saliva voluntarily all at once [38].

For each level and session that the participants played, the FitLab Game calibrated a baseline iHR, which allowed the game’s character to be placed in the center of the screen, in such a way that the starting point and the difficulty of the iHR series were equal for all participants. Then, participants had to follow the same pre-established route corresponding to each level and session, collecting apples (Bonus), which marked the correct route, and avoiding other elements such as unhealthy foods, tobacco, or the upper and lower limits marked by the floor and ceiling of the screen. To follow the route (or avoid certain elements), the main character had to move up or down the screen, which was controlled by changes in iHR. Participants in the control group were given no instructions on how to do so, whereas participants in the experimental group were explained the techniques mentioned in the paragraph above. All participants started the game with 20 lives, and the game ended when they ran out of lives or when they reached the end of the level without losing all these lives.

#### Data Analysis

Descriptive statistics are reported as means and SDs. A 2 × 2 multivariate analysis of variance following a general linear model was used to analyze the differences between the baseline and test situations. The baseline values were calculated as the average of the 2 games played in part 1, and the test values were calculated as the average of the 2 games played in part 3 (Figure 6). This statistical analysis was carried out to analyze the baseline versus test data obtained from HRV parameters, as well as time and Bonus, all separately. Statistical analysis was performed using SPSS (version 28.0; IBM Corp).

The raw RR interval values recorded by the Polar H10 cardiac chest band were processed to remove artifacts owing to false positive or false negative detections; afterward, the RR intervals were filtered, and the corresponding iHR values were shown in real time on the screen when the participant was playing. The same processed RR interval values were saved and analyzed to calculate the HRV parameters. A time domain HRV analysis was performed to calculate average RR intervals (RR mean), SD of RR intervals, root mean square of differences between adjacent RR intervals (RMSSD), and percentage of consecutive RR intervals that differ by >50 ms between them. A frequency domain analysis was used to calculate the low-frequency band (low frequency [LF]; 0.04-015 Hz) and high-frequency band (high frequency [HF]; 0.15-0.4 Hz). HRV analysis followed the guidelines recommended by the Task Force of the European Society of Cardiology and the North American Society of Pacing and Electrophysiology (1996). HRV analysis was performed using MATLAB (R2021 update 3 for 64 bits Windows; MathWorks).

### Ethics Approval

This study was conducted according to the local ethics committee for human experimentation (protocol code CEEAH-5745).

## Results

### Change in Gaming Performance (Pilot Study)

To analyze the participants’ performance in the game, the change in the number of apples collected (Bonus) and the total time played in seconds (Time) were considered, as presented in Table 2. No significant changes were found in the baseline versus test situation when comparing the experimental and control groups. However, there was a tendency for both groups to have increased Bonus and Time from the baseline to the test condition.

**Table 2:** Game outcomes in baseline and test phases in the control and experimental groups.

Variables			Baseline, mean (SD)		Test, mean (SD)
**Bonus**					
	Control	16.75 (10.91)		18.06 (9.54)	
	Experimental	15.75 (5.34)		20 (10.15)	
**Time**					
	Control	91.08 (26.79)		109.05 (58.17)	
	Experimental	91.07 (26.79)		116.67 (52.31)	

### Participants’ Satisfaction

The satisfaction questionnaire was completed by 13 (81%) of the 16 participants. Overall, participants expressed high satisfaction with the FitLab Game, reporting positive experiences, with a total score of 3.87 (SD 0.42) for the control group and a total score of 4.30 (SD 0.50) for the intervention group. The intervention group answered 16 questions, with 4 specific questions related to the techniques used during the game. Table 3 provides an extended description of the items and their scores for each group.

**Table 3:** Items and scores of the ad hoc game’s satisfaction questionnaire for the control group (n=5) and intervention group (n=8)^a^.

	Items	Control group, mean (SD)	Intervention group, mean (SD)
1	“I can learn self-control techniques better and faster with games like this.”	N/A^b^	4 (0.53)
2	“Using games like this would be easy for me.”	4.2 (0.45)	4 (1.07)
3	“Playing to learn techniques is more fun than following a pacer.”	4.6 (0.55)	4.87 (0.35)
4	“I would be able to play this game even if nobody was there to teach me.”	N/A	3 (0.76)
5	“In the future, I would play if I wanted to learn how to control my heart rate.”	4.2 (0.84)	4 (0.76)
6	“The aim of the game was clear the whole time I was playing.”	4 (1.22)	5 (0)
7	“The instructions for functionality were clear.”	N/A	5 (0)
8	“While playing, I understood how to achieve points and how to end the game successfully.”	4.2 (1.30)	4.6 (0.52)
9	“While playing, I was only thinking about the game.”	3.6 (1.14)	4.75 (0.46)
10	“While playing, I forgot everything else around me.”	4 (1)	4.5 (0.53)
11	“While playing, I didn’t realise how much time was passing.”	3.2 (0.84)	4.13 (0.83)
12	“When I did something in the game, I knew if it was right or wrong.”	3.4 (1.82)	4.25 (1.04)
13	“By playing I learned how the techniques affect my heart rate, and how I can modify it.”	N/A	4.13 (0.64)
14	“The game format increased my motivation to continue playing and achieve more bonus points.”	3.4 (0.89)	4.63 (0.52)
15	“A game that applied different breathing techniques would increase my interest in this topic.”	4 (0.71)	4.63 (0.52)
16	“The levels are fun and esthetically pleasing.”	4.2 (0.45)	4.38 (0.74)

^a^Total score: control group—mean 3.87 (SD 0.42); intervention group—mean 4.30 (SD 0.50).

^b^N/A: not applicable.

### Change in HRV Parameters

**Figure 7:**
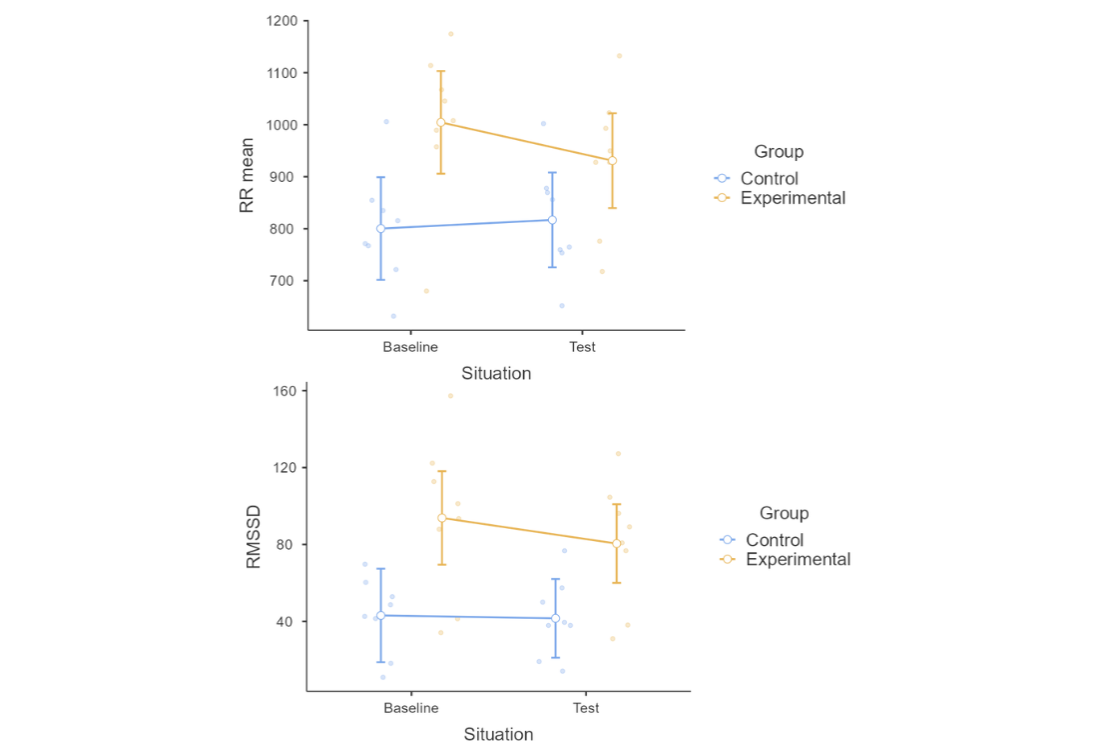
Mean, 95% CI, and distribution of average RR intervals (RR mean) and root mean square of differences between adjacent RR intervals (RMSSD), when comparing baseline and test situations in the experimental and control groups.

When analyzing the changes in HRV before and after the game, the results showed some differences between the experimental and control groups (Table 4). In particular, the experimental group showed a decrease in RR mean from baseline to test, which was different from the increase observed in the control group (*P*=.02; Figure 7). In contrast, the experimental group showed a decline in RMSSD from baseline to test, which was more pronounced than that in the control group (*P*=.04). No other HRV parameters presented a significant or tendential change.

**Table 4:** Multivariate analysis of variance of heart rate variability variables in baseline and test conditions in the control and experimental groups.

Variables		Baseline, mean (SD)	Test, mean (SD)	*P* value	
**RR mean^a^**					.02
	Control	800.33 (109.02)	816.89 (106.59)		
	Experimental	1004.52 (148.41)	930.90 (132.45)		
**SDRR^b^**					.56
	Control	62.55 (24.12)	65.64 (20.16)		
	Experimental	111.35 (33.97)	109.81 (32.44)		
**RMSSD^c^**					.04
	Control	43.10 (19.96)	41.60 (20.16)		
	Experimental	93.80 (40.71)	80.07 (31.73)		
**pNN50^d^**					.36
	Control	23.83 (17.53)	20.77 (17.25)		
	Experimental	42.90 (18.63)	34.64 (17.52)		
**LF^e^**					.39
	Control	3063.58 (2789.61)	2595.49 (2190.80)		
	Experimental	6612.13 (3438.65)	6972.52 (3760.22)		
**HF^f^**					.10
	Control	661.70 (458.82)	739.14 (547.80)		
	Experimental	2985.17 (2311.75)	2046.96 (1594.17)		

^a^RR mean: average RR intervals.

^b^SDRR: SD of RR intervals.

^c^RMSSD: root mean square of differences between adjacent RR intervals.

^d^pNN50: percentage of consecutive RR intervals that differ by >50 ms between them.

^e^LF: low frequency.

^f^HF: high frequency.

## Discussion

### Overview

The aim of this study was 2-fold: to develop a serious game that teaches the user to self-regulate iHR using HRVB and to carry out a pilot study to test the game with participants. A total of 16 participants took part in the pilot study and were divided into a control group and an experimental group. The results were analyzed in 2 parts: one focused on changes in the performance of the game and the other on fluctuations on HRV parameters. The results related to the usefulness of the game are discussed in the *Game and App Performance* section.

### Game and App Performance

Analyzed globally, the gaming performance of all participants was in line with the results of another gaming study [39], in which the participants played a biofeedback game 6 times and both the control group that received simulated feedback and the experimental group that received physiological feedback improved their gaming performance. These results indicate that playing a given game, in itself, leads to improvement in its final score through repeated training or habituation. That is, both groups got to experiment and learn how to move the game’s character. Regarding the differences in performance between groups, the results showed that the experimental group had a greater improvement in all indicators compared with the control group, even though the differences were not significant. The experimental group showed a greater improvement in Bonus score (increase of 4.25 Bonus points in the test with respect to the baseline), compared with the control group (increase of 1.31 Bonus points in the test; Table 2). The same trend was observed in the total playing time, where the experimental group kept playing 7.6 seconds longer than the control group, but these results were not statistically significant (Table 2). All these results are consistent with the idea that the application of specific strategies in the experimental group could have improved the self-regulation of heart variability, more than simply practicing the game (as shown with the control group). However, these results should be interpreted with caution because of the small sample size and the high values of SDs shown in Table 2.

In our study, a few gamification techniques were introduced to increase players’ desire to engage with the tasks. General techniques include having a goal to aim toward, a set of rules to follow, a challenge to complete, and *real*-*time* feedback to tell the player how he is doing and how close he is to completing the goal. More specific gamification techniques included Bonus points, increasing challenges, and performance graphs. Reynard et al [40], in a systematic review and meta-analysis of digital interventions for emotion regulation, found that digital game interventions consistently provided stronger evidence compared with other types of digital interventions. In this review, 64% of digital game studies provided feasibility for engagement, implementation, adherence, expectations, and transference to real life. However, they did not explain which elements of the specific game design were responsible for this feasibility. For their part, Maher et al [41] concluded that gamified apps were used substantially longer than nongamified apps. Other studies highlight specific game elements that impact health behavior and achieve adherence, similar to digital rewards, such as points, badges, or rewards that we used in our game [42]. Similarly, in the design of our game, we complied with five gamification principles relating to the motivation of the user [43,44]: (1) meaningful purpose, as the goal of the users participating in the study was to learn a technique to improve their health; (2) meaningful choice, as users could control how they achieved their goal of eating the apples on the screen; (3) supporting player archetypes, as in our game, the initial iHR level is individualized based on the user characteristics; (4) feedback, as the user’s actions to control the movement on the screen based on their own cardiac variability affect progress, and it is clearly communicated; and (5) visibility, as the amount of progress made in each level (apples, points, and Bonuses achieved) is visible at all times. Most of these principles are reaffirmed by the users’ responses to the subsequent questionnaire, as discussed in *Participants’ Satisfaction* section. Thus, the different techniques applied in the present serious game could contribute to behavioral changes, such as increasing players’ engagement with the tasks and adhering to the use of the game. For example, the instant feedback provided by the game may have helped produce high engagement and enjoyability for the participants, as noted by Siriaraya et al [45]. At the same time, the fact that the levels of the game are adapted to each participant’s cardiac recording means that each person plays in a personalized environment, which is thought to increase motivation toward the game [46]. Finally, regarding the app’s functionality, we highlight the possibility of using either Bluetooth devices or photoplethysmography sensors [47], allowing participants to play in different contexts and with different resources.

The serious game created for this study can be used in multiple situations (in the long term). The game provides flexibility to play in new scenarios, depending on the specific training or situation goals set by the player. Furthermore, there is a menu for practice in which the players can play to test different techniques. Therefore, this game supports repetitive practice as an effective training method. In addition, the game uses the Bonus and time measurements to self-track the learning progression. As the players enhance their level of expertise, the Bonus points and time increase accordingly. This mechanism ensures that as the player improves, they are rewarded with higher Bonuses and more time in the level, motivating them.

### Participants’ Satisfaction

Participants expressed an overall positive satisfaction with the FitLab Game, with a greater total score in the intervention group (4.30 out of 5) than in the control group (3.87 out of 5). As Table 3 shows, this higher satisfaction in the intervention group is also supported by the fact that all the items that both groups had in common showed a higher score of 4 (out of 5) only for the intervention group. Both groups considered the game to be easy and fun (punctuations >4 for both groups). The goal and strategic approach to achieve Bonuses and continue playing were clear for both groups, with scores >4. Furthermore, both groups expressed positive intention (score >4) to continue playing the FitLab Game and learn how to control their HR in the future. The participants in the intervention group rated the feedback provided by the game more positively (score >4) compared with the control group (score >3). Regarding the experience of flow during the game, although both groups scored >3, the intervention group scored >4 across the 3 items. Similarly, in terms of motivation, the intervention group scored >4 and the control group scored >3. Finally, both groups found the screens visually appealing and balanced in terms of colors, with scores >4.

As shown in Table 3, the techniques provided to the intervention group were found to be useful in establishing control over iHR and were considered important for playing successfully. Thus, the neutral score in the personal ability factor indicates that the instructions based on different techniques for controlling iHR should be considered necessary for success in the game. The gameplay was understood by both the groups, and the game was generally perceived as easy and enjoyable. With regard to flow and motivation, the intervention group reported a more favorable experience with the game. This can be explained by the use of techniques that enable participants to exert voluntary control over the characters, thereby enhancing the game experience through the interactive component of the game. These findings are in line with those of previous studies, indicating that a gamified visualization component results in higher intrinsic motivation [48]. It is worth noting that the participants expressed a strong interest in continuing to play the FitLab Game in the future, reflecting their engagement and willingness to play to learn about cardiac variability control.

On the basis of the results presented, we propose an extensive use of the game with the main goal of helping individuals learn how to self-regulate their cardiac variability to achieve emotional self-control. This could be applied in different contexts, including clinical (eg, in anxiety or stress disorders) and nonclinical populations. This is in line with the results of a systematic review where consistent evidence was found for reduced negative emotional experience provided by digital games [40]. Ideally, these games should be used under the supervision of health professionals to facilitate learning-specific techniques for controlling cardiac variability. This is reaffirmed because the only response <4 in the specific items for the intervention group is for the item: “4 I would be able to play this game even if nobody was there to teach me” (rating of 3 out of 5). Nonetheless, as shown in Table 3, participants reported that the game was easy to understand and play (item 2), indicating that both the control and experimental groups understood the game well.

### HRV Analysis and Biofeedback

Regarding HRV, the results of the pilot study showed some differences in the cardiac behavior of the experimental and control groups (Table 4; Figure 7). This suggests that the strategies used by the experimental group to compete in the game improved their control of cardiac variability, which in turn may have enhanced their performance. Owing to its pilot nature and limited number of participants, the precise reasons underlying the relationship between control of cardiac variability and performance cannot be conclusively determined. Nonetheless, it is possible that the results reflect the activation and the control of attention mechanisms in the experimental group. If we consider that the game requires a certain level of concentration and attention to play, especially if the participants are remembering and applying the techniques that were just learnt, some arousal level is needed, reflected in a reduction in the activity of the parasympathetic nervous system, as shown by a reduction, although not significant, in the experimental group (HF parameter) compared with a slight increase in the control group. This result is consistent with the increase, but not significant, in the LF values in the experimental group compared with the decrease in the control group. There is evidence that higher LF values are related to a greater activation of the sympathetic nervous system [49]. At the same time, a significant reduction in cardiac variability was observed in the experimental group, as shown by the RR mean and RMSSD parameters. The set of previous results could explain the improvement in the control of arousal or activation in the experimental group. In line with this idea, Fuentes-García et al [50] found that in chess games, the player’s level and the difficulty of the game determined the activity of the ANS of the individuals, with higher demands in attentional focus resulting in lower HRV. Thus, in this study, the experimental group may have shown reduced vagal activity and cardiac variability because they were given techniques to control their HRV, requiring greater attention when playing [51]. In that sense, HRV is an indicator that the experimental group was active and applying what had been taught.

An array of scientific papers highlights the potential of incorporating biofeedback into different types of digital game interventions, including virtual and augmented reality, internet therapy, biofeedback and neurofeedback, digital games, and web-based programs. In a systematic review by Reynard et al [40], 39 interventions were identified, 10% of which were based on biofeedback. The effectiveness and impact of incorporating biofeedback into digital games have been tested in a range of topics, from stress reduction to physical rehabilitation [52]. For instance, Knox et al [53] and Wenck et al [54] studied biofeedback-driven digital game interventions for stress levels before the emergence of smartphones and concluded that symptoms of anxiety and depression were significantly alleviated in children. Similarly, Jerčić and Sundstedt [55] showed that visual and gameplay biofeedback in serious games supported emotional regulation skills. Moreover, aside from the impact on the psychophysiology of individuals, digital interventions appear to be accessible and attractive compared with one-to-one clinical therapies [40], particularly for the younger population [56]. Lüddecke and Felnhofer [57] demonstrated that biofeedback interventions increased the motivation and long-term participation of users in health-related interventions. Finally, games that incorporate biofeedback, compared with other digital interventions, seem to provide the most evidence for emotional skills transference to real life [40]. In conclusion, there is a growing body of research that emphasizes the utility of incorporating biofeedback into digital game interventions for its attractiveness and potential to enhance emotional regulation, among other benefits.

Returning to our study, our results seem to contradict most studies using the HRVB technique, which conclude that this technique helps improve HRV [58,59] with the general aim of achieving relaxation. However, this was not the case. These articles used set breathing paces, usually between 4.5 and 6.5 times per minute, as HRVB to induce relaxation [60,61]. For example, on the topic of gaming, Al Osman et al [62] used game-based biofeedback to control the activity of the ANS and showed that HRV increased following a controlled breathing rate. In contrast, the goal of this study was not to increase, maintain, or decrease HRV but to teach participants to control its rises and falls through various techniques, including breathing control, but not at a set pace. Thus, our study is not comparable with most other studies, given that we did not focus on breathing paces but rather on an array of techniques to teach the participants how to increase or decrease their HRV. These techniques include the voluntary production of apneas to suddenly increase cardiac variability [35], the performance of muscle movements to achieve a reduction in HRV (in line with Guidi et al [37]), or voluntary swallowing. This last strategy can produce changes in some HRV parameters in healthy people, such as SD of RR intervals, LF, and HF power [63], and effortful swallowing increases LF power and the LF/HF ratio [64]. On a practical level, in our case, these strategies allowed the player to move the character up and down the game screen because they provide rapid changes in cardiac variability.

### Limitations and Future Directions

This study had 3 main limitations. The first limitation is the sample size of the pilot study. Even if the results show significance in the mean iHR (proportional to the RR mean) and RMSSD, they should still be considered preliminary. The second limitation is that participants were not monitored before and after the game but only during it. This means that the HRV indicators were collected as the game was being played, thus in an active state and not in a resting situation. Our interest was to test whether there were changes in the HRV parameters and game performance at all, as a pilot study, but in future studies, it would be interesting to observe the influence of our intervention on basal HRV.

Finally, most previous studies using techniques such as HRVB included a sample of clinical patients, unlike this study, which was conducted in healthy university students. For instance, Siepman et al [65] compared the effects of HRVB in both healthy people and individuals classified as depressed, showing that the latter experienced a significantly greater improvement in HRV, compared with healthy volunteers. Similarly, Pyne et al [66] reported that HRVB training reduces symptoms of posttraumatic stress in older people only. These studies highlight that HRV parameters seem to be difficult to alter with HRVB in healthy and younger people than in older or clinical populations, potentially explaining the results of this study, with few to no significant changes in some HRV parameters. In the future, a larger sample size of healthy university students or testing the FitLab Game in a clinical population could provide more consistent results. Finally, it would also be interesting to provide new strategies for the voluntary control of HRV, perhaps further refining the ones we have already developed in this study. It would also be necessary to deepen the swallowing strategy, as Yildiz and Doma [38] and Yıldız [67] concluded that spontaneous saliva swallowing can change some short-term HRV parameters significantly even in healthy people.

### Conclusions

This study developed a serious game, called the FitLab Game, that teaches the user to self-regulate the iHR using cardiac variability biofeedback. The game was also tested with the participants in a pilot study. Overall, the FitLab Game appears to be a promising serious game because of its ease of use, high engagement, and enjoyability provided by the instantaneous feedback. The strategies learned by the experimental group to compete in the game, which included the voluntary production of apneas, muscle movements, and voluntary swallowing, improved the self-control of cardiac variability and, in turn, may have enhanced performance. A significant reduction in cardiac variability observed in the experimental group, based on the RR mean and RMSSD parameters, could explain the improvement in the management of arousal or activation compared with the control group. Thus, the application of specific strategies in the experimental group may have improved the self-regulation of HRV more than simply practicing the game.

The usefulness of HRV as a health indicator is well known, and HRV biofeedback is considered an established intervention in the clinical context. Thus, learning simple and voluntary strategies through the developed game can help improve self-control and arousal management. Ultimately, with sufficient training, the game can become a relaxation technique for the general population or clinical populations, or, for example, an activation technique for athletes in certain situations.

## Appendix

Multimedia Appendix 1 Gameplay of the FitLab Game.

## Abbreviations

ANS: autonomic nervous system
HF: high frequency
HR: heart rate
HRV: heart rate variability
HRVB: heart rate variability biofeedback
iHR: instantaneous heart rate
LF: low frequency
RMSSD: root mean square of differences between adjacent RR intervals
RR mean: average RR intervals
